# Plasma Soluble Urokinase-Type Plasminogen Activator Receptor Level as a Predictor of the Severity of Community-Acquired Pneumonia

**DOI:** 10.3390/ijerph16061035

**Published:** 2019-03-21

**Authors:** Ping-Kun Tsai, Shih-Ming Tsao, Wei-En Yang, Chao-Bin Yeh, Hsiang-Ling Wang, Shun-Fa Yang

**Affiliations:** 1 Institute of Medicine, Chung Shan Medical University, Taichung 40201, Taiwan; pingkun@hotmail.com (P.-K.T.); weienyang@gmail.com (W.-E.Y.); 2 Department of Internal Medicine, Zuoying Branch of Kaohsiung Armed Forces General Hospital, Kaohsiung 813, Taiwan; 3 Institute of Biochemistry, Microbiology and Immunology, Chung Shan Medical University, Taichung 40201, Taiwan; tsmhwy@ms24.hinet.net; 4 School of Medicine, Chung Shan Medical University, Taichung 40201, Taiwan; sky5ff@gmail.com; 5 Division of Chest, Department of Internal Medicine, Chung Shan Medical University Hospital, Taichung 40201, Taiwan; 6 Department of Medical Research, Chung Shan Medical University Hospital, Taichung 40201, Taiwan; 7 Department of Emergency Medicine, Chung Shan Medical University Hospital, Taichung 40201, Taiwan; 8 Department of Beauty Science, National Taichung University of Science and Technology, Taichung 404, Taiwan; charmaine790520@yahoo.com.tw

**Keywords:** community-acquired pneumonia, suPAR, LPS, pneumonia severity index

## Abstract

The urokinase-type plasminogen activator receptor (uPAR) mediates various cellular activities and is involved in proteolysis, angiogenesis, and inflammation. The objective of this study was to investigate the association between soluble uPAR (suPAR) levels and community-acquired pneumonia (CAP) severity. A commercial enzyme-linked immunosorbent assay (ELISA) was performed to measure the plasma suPAR levels in 67 healthy controls and 75 patients with CAP. Our results revealed that plasma suPAR levels were significantly elevated in patients with CAP compared with the controls, and antibiotic treatment was effective in reducing suPAR levels. The plasma suPAR levels were correlated with the severity of CAP based on the pneumonia severity index (PSI) scores. Furthermore, lipopolysaccharide (LPS)-stimulation significantly increased uPAR expression in RAW 264.7 macrophages. In conclusion, plasma suPAR levels may play a role in the clinical assessment of CAP severity; these findings may provide information on new targets for treatment of CAP.

## 1. Introduction

The urokinase-type plasminogen activator receptor (uPAR), a multi-ligand receptor, can bind to various ligands such as urokinase-type plasminogen activator (uPA), integrins, and vitronectin [1]. uPAR mediates various cellular activities and is involved in cell migration, cell adhesion, proteolysis, angiogenesis, and inflammation [2,3,4,5,6].

Recently, the soluble form of the urokinase-type plasminogen activator receptor (suPAR) has been proposed to be a biomarker in various inflammatory or immune diseases, including sepsis, diabetes mellitus, and systemic lupus erythematosus (SLE) [7,8]. Several studies have described its potential for the prediction of various types of pneumonia [9,10]. Wrotek et al. reported a correlation between higher suPAR and longer hospitalization of children with community-acquired pneumonia (CAP) [9]. Savva and colleagues investigated suPAR levels in 180 patients with ventilator-associated pneumonia (VAP) and showed that serum suPAR is an independent predictor of unfavorable outcome in VAP [10]. The associations of suPAR with markers of inflammation have been studied in part in various types of pneumonia, but little is known about these associations in community-acquired pneumonia. 

Community-acquired pneumonia (CAP) is the most common infectious disease with high mortality [11,12]. The CAP mortality rate remains high despite numerous treatment options. Therefore, recognition of the severity and early diagnosis of CAP is crucial. Although previous studies have reported the potential role in various types of pneumonia, the correlation of suPAR levels with the severity of pneumonia remains unclear. The aim of this study was therefore to investigate a potential association between the concentrations of plasma suPAR and the effectiveness of an antibiotics treatment in CAP.

## 2. Materials and Methods

### 2.1. Subjects

The study groups comprised 67 healthy controls (45 males and 22 females; with a mean age of 61.1 ± 10 years) and 75 community-acquired pneumonia patients (52 males and 23 females; with a mean age of 65.5 ± 17.7 years) recruited from Chung Shan Medical University Hospital (CSMUH) (Taichung, Taiwan). The diagnostic criteria for CAP were based on guidelines of the American Thoracic Society (ATS). The study protocol was approved by the Institutional Review Board of Chung Shan Medical University Hospital (CSMUH No.: CS11237), and informed written consent was obtained from each individual before initiation of the study. Blood samples were collected before patients with CAP received treatment protocols, and post-treatment blood samples were obtained (median time of treatment: 3 days) after the pneumonia had resolved. Venous blood from each participant was taken in an EDTA-containing tube, immediately centrifuged, and then stored at −80 °C.

### 2.2. Measurement of Plasma suPAR

An enzyme-linked immunosorbent assay (ELISA) was used to measure concentrations of suPAR in plasma samples of control and CAP patients (Catalog Number DUP00, R&D Systems Inc., MN, USA) as previously described [4]. For each sample, 50 μL plasma samples were added to microtest strip wells for 2 h at room temperature. The antibody binding was detected with streptavidin-conjugated horseradish peroxidase after four washing steps. Subsequently, the reaction was stopped, and the optical density was determined with a microplate reader set to 450 nm. suPAR concentrations were calculated from a standard curve. 

### 2.3. Cell and Cell Culture

The RAW 264.7 macrophage cell lines were purchased from ATCC (Manassas, VA, USA). The RAW 264.7 macrophage cell lines was cultured in Dulbecco’s modified Eagle’s medium with 10% fetal bovine serum as previously described [13]. All cell cultures were maintained at 37 °C in a humidified atmosphere of 5% CO_2_.

### 2.4. Western Blot Analysis

Cells were lysed in Radio Immuno Precipitation Assay (RIPA) buffer containing protease inhibitors as described previously [14]. Equal amounts of proteins were separated by 10% SDS-PAGE and transferred to nitrocellulose paper, after blocking with 5% non-fat milk in Tris-buffered saline and then overnight with polyclonal antibodies against suPAR (R&D Systems Inc., Minneapolis, MN, USA) and β-actin (Abcam, Cambridge, UK). Afterwards, signals were detected by using enhanced chemiluminescence (ECL) commercial kit (Millipore, Burlington, MA, USA).

### 2.5. Immunofluorescence Staining

For immunofluorescence staining, the RAW 264.7 macrophage cell lines were seeded onto coverslips in six-well plates (2 × 10^5^ cells/well) and then treated with lipopolysaccharide (LPS; 0.5 μg/mL) for 24 h and then washed with 1 × cold PBS twice as previously described [15]. The results were analyzed by a confocal microscope.

### 2.6. Statistical Analysis

The number (*n*) was expressed as percentages for categorical variables and all continuous variables are expressed as the mean ± SD. The Mann–Whitney U-test was performed to compare between untreated patients and healthy individuals. For correlations of suPAR with PSI scores of CAP patients, the linear regression analysis was performed. A *p* < 0.05 was considered statistically significant. Statistical analyses were performed using SPSS (version 17, SPSS, Chicago, IL, USA).

## 3. Results

The demographic and clinical characteristics of the participants are summarized in Table 1. The age and gender of the participants did not significantly differ between the 75 CAP patients and the 67 individuals in the control group (*p* = 0.075 and *p* = 0.782, respectively). For the clinical characteristics, patients with CAP had significantly higher C-reactive protein (CRP) levels (12.41 ± 7.85 vs. 0.49 ± 0.27 mg/dL), white blood cells (WBCs; 13,211.2 ± 6375.4 vs. 6259.6 ± 2025.1 cells/mm^3^), and neutrophils (10,505.3 ± 5248.8 vs. 3736.4 ± 1526.8 cells/mm^3^) compared to control subjects (*p* < 0.001). Furthermore, there were significant decreases in CRP concentrations (untreated: 12.41 ± 7.85 mg/dL; treated: 3.84 ± 4.01 mg/dL), WBCs (untreated: 13,211.2 ± 6375.4 cells/mm^3^; treated: 10,085.5 ± 5251.7 cells/mm^3^), and neutrophils (untreated: 10,505.3 ± 5248.8 cells/mm^3^; treated: 7548.3 ± 4165.9 cells/mm^3^) after antibiotic treatment (*p* < 0.001) (Table 1). 

**Figure 1:**
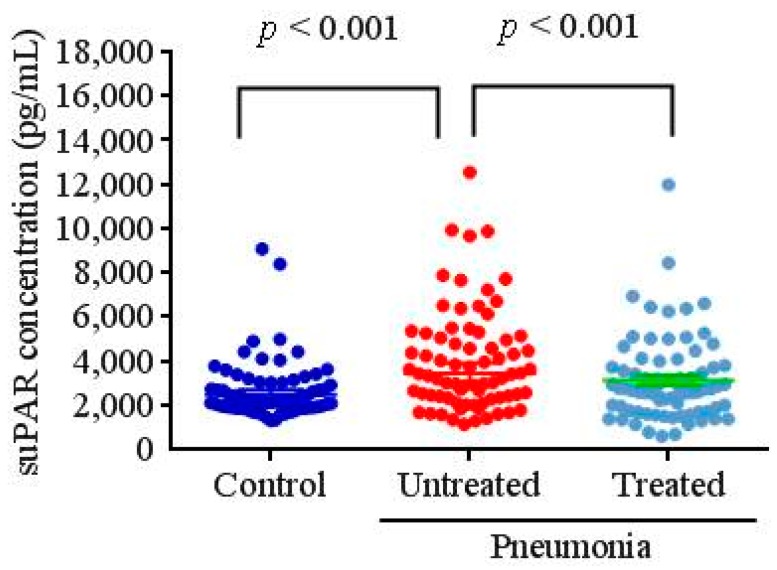
Plasma concentrations of soluble urokinase-type plasminogen activator receptor (suPAR) in control subjects and patients with community-acquired pneumonia (CAP) before and after treatment.

Figure 1 shows plasma suPAR concentrations in control subjects and CAP patients before and after antibiotic treatment. As shown in Figure 1, patients with CAP had significantly higher plasma suPAR levels compared to control subjects (controls: 2673.0 ± 1369.6 pg/mL; patients: 3980.5 ± 2315.1 pg/mL; *p* < 0.001). After CAP patients received antibiotic treatment, suPAR concentrations significantly decreased (untreated: 3980.5 ± 2315.1 pg/mL; treated: 3113.9 ± 1920.7 pg/mL; *p* < 0.001).

**Figure 2:**
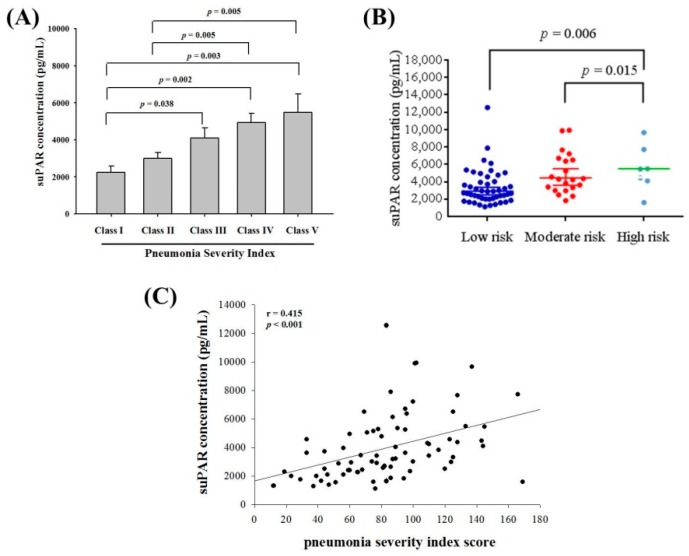
Correlations of plasma concentrations of soluble urokinase-type plasminogen activator receptor (suPAR) with the pneumonia severity index (PSI) and mortality risk in 75 patients with community-acquired pneumonia (CAP). (**A**) Significant differences were observed between different PSI class scores. Data are expressed as the mean ± standard deviation. (**B**) Plasma suPAR concentrations were significantly higher in CAP patients with high mortality risk than patients with moderate or low mortality risk. (**C**) A significantly positive correlation was observed between plasma suPAR levels and PSI scores (Spearman’s correlation coefficients: *r* = 0.415, *p* < 0.001).

To further examine the correlation between plasma suPAR concentrations and the severity of CAP before treatment, the pneumonia severity index (PSI) was used. As shown in Figure 2A, significant differences in suPAR concentrations were observed between Class I and Class III, Class IV as well as Class V patients. Moreover, as shown in Figure 2B, suPAR concentrations were higher in high risk patients than those with low risk patients (*p* = 0.006). Furthermore, a correlation was detected between suPAR levels and PSI scores (*r* = 0.415; *p* < 0.001; Figure 2C).

**Figure 3:**
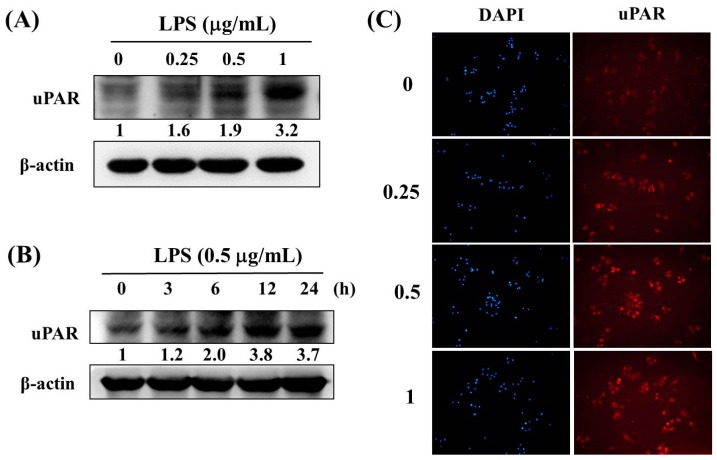
Effect of lipopolysaccharide (LPS) on the protein level of uPAR in Raw 264.7 cell lines. (**A**) Raw 264.7 cells were treated with the vehicle or LPS (0.25–1 μg/mL) for 24 h and then subjected to a western blot analysis. Quantitative uPAR protein levels were adjusted to the β-actin protein level. (**B**) Raw 264.7 cells were treated with LPS (0.5 μg/mL) for 0, 3, 6, 12, or 24 h and then subjected to a western blot analysis. Quantitative uPAR protein levels were adjusted to the β-actin protein level. (**C**) Raw 264.7 cells were treated with LPS (0.25–1 μg/mL) for 24 h and the uPAR expression was performed by immunofluorescence under confocal microscopy.

According to previous studies, LPS-treated RAW 264.7 macrophages is a reliable model to mimic the bacterial pneumonia in vitro [16,17]. Thus, western blotting assays were used to investigate the expression of uPAR protein expression after LPS treatment of RAW 264.7 cells. As shown in Figure 3A and 3B, the results revealed that LPS significantly induced uPAR protein in a concentration-dependent manner (Figure 3A) and in a time-dependent manner (Figure 3B) in the RAW 264.7 cells. Moreover, exposure to 1 μg/mL LPS for 24 h significantly increased the uPAR expression in the RAW 264.7 cell line by using immunofluorescence staining (Figure 3C).

## 4. Discussion

Soluble urokinase-type plasminogen activator receptor (suPAR) results from the division of urokinase-type plasminogen activator receptor (uPAR) by various factors, which is then released into the blood [1,4,16]. Therefore, suPAR can be used to diagnose numerous diseases [5,8,17,18]. This study demonstrated that suPAR levels were elevated in CAP patients and significantly decreased in the same patients after they received antibiotic treatment. The plasma suPAR concentrations in the CAP patients were associated with PSI scores indicative of the disease severity. These results were consistent with those of Wrotek et al. relating the correlation between plasma suPAR levels and CAP severity [9].

Numerous studies have adopted the pneumonia severity index and the CURB-65 (confusion, urea nitrogen, respiratory rate, blood pressure, 65 years of age and older) to assess the mortality and severity of community-acquired pneumonia (CAP) [19,20,21,22]. However, these indices can only be obtained through the collection of a substantial amount of clinical and examination data. Hence, numerous studies have investigated the feasibility of using relatively convenient biomarkers such as white blood cell count, C reactive protein, and procalcitonin as tools to predict CAP severity [23,24,25]. Although white blood cell count and C reactive protein have been applied to numerous clinical examinations, they are still incapable of estimating CAP severity and mortality risk. Because we determined a correlation between suPAR and CAP severity, we attempted to evaluate the feasibility of using suPAR as a cofactor for the estimation of CAP severity and mortality. 

In addition to clinical specimens, lipopolysaccharide (LPS)-induced macrophages were also examined in this study. The results revealed that LPS significantly induced uPAR protein in the RAW 264.7 cells. RAW 264.7 cells are the most widely adopted cell line in in vitro experiments; they can express and release numerous cytokines through LPS stimulation [26,27,28,29]. Furthermore, Koch et al found that under LPS stimulation, monocytes quickly divided uPAR to transform it into suPAR [30]. We also used this model to confirm the importance of suPAR for determining pneumonia severity. Apart from in vitro cell experiments, the literature indicated that in animal experiments suPAR level was specifically increased, while uPAR was rapidly cleaved from monocytes by lipopolysaccharide (LPS) stimulation [30]. This result is consistent with the data we obtained from clinical specimens and cell experiments. Furthermore, other researchers have conducted experiments with uPAR-deficient mice and determined that that these mice could not overcome the immune response caused by *Pseudomonas aeruginosa*-induced pneumonia [31]. Thus, in addition to serving as an auxiliary factor for the pneumonia severity, suPAR may serve as an auxiliary tool for pneumonia treatment in the future. Once the price of suPAR kits is affordable to patients and clinical applications, they could be a practical option in the clinical treatment of pneumonia.

A limitation of our study is that we lacked data regarding renal function for our study groups, so the independent association between suPAR and pneumonia severity could not be established. Moreover, more information from CAP patients, non-pneumonia infectious diseases patients, as well as ill patients without infections should be collected for further investigation, which will be included in our future work.

## 5. Conclusions

In conclusion, our study provided important information regarding the plasma concentrations of suPAR in the assessment of CAP severity, which may be used for predicting CAP severity in Taiwanese populations. These results suggested that detecting plasma suPAR expression can be useful in the clinical management of CAP.

## Figures and Tables

**Table 1:** Laboratory data of both controls and patients with community-acquired pneumonia (CAP) before and after they received treatment.

Clinical Variable	Controls (*n* = 67)	Before Antibiotic Treatment (*n* = 75)	After Antibiotic Treatment (*n* = 75)	*p* Value UT/C	*p* Value UT/T
Age	61.10 ± 10.13	65.52 ± 17.71		*p* = 0.075	
Gender					
Male	45 (67.2%)	52 (69.3%)		*p* = 0.782	
Female	22 (32.8%)	23 (30.7%)			
CRP (mg/dL)	0.49 ± 0.27	12.41 ± 7.85	3.84 ± 4.01	*p* < 0.001	*p* < 0.001
WBCs (cells/mm^3^)	6259.6 ± 2025.1	13211.2 ± 6375.4	10085.5 ± 5251.7	*p* < 0.001	*p* < 0.001
Neutrophils (cells/mm^3^)	3736.4 ± 1526.8	10505.3 ± 5248.8	7548.3 ± 4165.9	*p* < 0.001	*p* < 0.001
PSI score					
I		10 (13.3%)			
II		16 (21.4%)			
III		21 (28.0%)			
IV		21 (28.0%)			
V		7 (9.3%)			

CRP, C-reactive protein; WBCs, white blood cells; PSI, pneumonia severity index; C, controls; UT, patients with CAP before they received antibiotic treatment; T, patients with CAP after they received antibiotic treatment.
